# Flowchart for selecting an appropriate surgical airway in neurologically impaired pediatric intubated patients: a case series^☆^

**DOI:** 10.1016/j.bjorl.2023.101290

**Published:** 2023-07-07

**Authors:** Mitsuyoshi Imaizumi, Kazuhide Suyama, Aya Goto, Mitsuaki Hosoya, Shigeyuki Murono

**Affiliations:** a Fukushima Medical University, School of Medicine, Department of Otolaryngology, Fukushima, Japan; b Fukushima Medical University, School of Medicine, Department of Pediatrics, Fukushima, Japan; c Fukushima Medical University, Health Information and Epidemiology Center for Integrated Science and Humanities, Fukushima, Japan

**Keywords:** Flowchart, Surgical airway, Tracheostomy, Neurologically impaired pediatric patient, Aspiration prevention surgery

## Abstract

**Objective:**

Medical advances have resulted in increased survival rates of neurologically impaired children who may require mechanical ventilation and subsequent tracheostomy as a surgical airway. However, at present, there is no definite consensus regarding the timing and methods for placement of a surgical airway in a neurologically impaired intubated child who needs to be cared for over a long-term period. We therefore created a flowchart for the selection of a surgical airway for Neurologically Impaired Pediatric Patients (NIPPs).

**Methods:**

The flowchart includes information on the patients’ backgrounds, such as intubation period, prognosis related to reversibility, and history of aspiration pneumonia. To evaluate the importance of the flowchart, first we conducted a survey of pediatricians regarding selection of a surgical airway, and we also evaluated the appropriateness of the flowchart among pediatricians and caregivers through questionnaire surveys which include satisfaction with the decision-making process, and postoperative course after discharge.

**Results:**

A total of 21 NIPPs with intubation underwent surgery and a total of 24 participants (14 pediatricians and 10 caregivers) completed the survey. The answers regarding the importance of the flowchart showed that eleven pediatricians had experience selecting of surgical airways, nine of whom had had experiences in which they had to make a difficult decision. The answers regarding the appropriateness of the flowchart revealed that all pediatricians and caregivers were satisfied with the decision-making process and postoperative course after discharge using the flowchart.

**Conclusions:**

The present study demonstrated the effectiveness of our flowchart for selecting an appropriate surgical airway in NIPP. By referring to our flowchart, pediatricians and caregivers are likely to be able to select an appropriate surgical airway, leading to increased satisfaction with the decision-making process and postoperative course.

**Level of Evidence:**

4.

## Highlights

• Survival rate of severely impaired children requiring tracheostomy is increasing.
• Appropriate surgical airway selection in intubated children is difficult.
• We designed a flowchart for surgical airway selection in intubated pediatric patients.
• Effectiveness of the flowchart for selecting a surgical airway was demonstrated.
• Pediatricians and caregivers will be able to select an appropriate surgical airway.

## Introduction

Over the last decade, in neonatal and pediatric ICU care, medical advances have resulted in increased survival of premature neonates, those with complex anomalies, and Neurologically Impaired Pediatric Patients (NIPP). Such patients sometimes require long-term mechanical ventilation and subsequent tracheostomy. Therefore, pediatric patients who receive tracheostomy have been increasing, and the overall result has been a general trend for patients who require tracheostomy to be younger and more likely to have chronic diseases.^1^, ^2^, ^3^

However, there is no definite consensus regarding the length of time a child should remain endotracheally intubated before tracheostomy is performed. Without defined criteria, tracheostomy is often decided solely by the medical team in charge of the patient. Therefore, for standardized indications, the guidelines for tracheostomy in patients in the ICU were established.^4^ However, these guidelines are not applicable to NIPP with prolonged intubation. The backgrounds of pediatric patients differ from those of adult patients, in terms of considerations such as future reversibility of a condition, possibility of long-term survival with impairments, and parent/caregiver intentions.

Another potential solution for NIPP with prolonged intubation is Laryngotracheal Separation (LTS), which is a type of Aspiration Prevention Surgery (APS) first described by Lindeman et al.^5^ Most children with irreversible conditions, such as neurological impairment, experience worsening of swallowing function, which can lead to death. Therefore, Hara et al. recommended LTS instead of tracheostomy for NIPP with indications of respiratory failure.^6^ However, it is difficult for the medical team alone to select an appropriate surgical airway in intubated NIPP without multidisciplinary involvement in the decision and/or referable criteria.

To solve these previously mentioned problems, we hypothesized that a method to select an appropriate surgical airway using a flowchart would provide many benefits, such as: easier selection by a pediatrician who is not familiar with the evaluation of laryngeal and tracheal function associated with swallowing impairment and aspiration; less invasiveness by avoiding multiple surgeries; more efficiency regarding time; less confusion in the decision-making process; and less burden on caregivers regarding the care of tracheostomized children.

The aims of the present study were to create a flowchart for selecting a surgical airway in NIPP with intubation and evaluate its appropriateness from the viewpoints of the satisfaction of both the attending pediatrician and caregivers about the process of surgery selection and the care after discharge.

## Methods

### Creation of the flowchart

**Figure 1:**
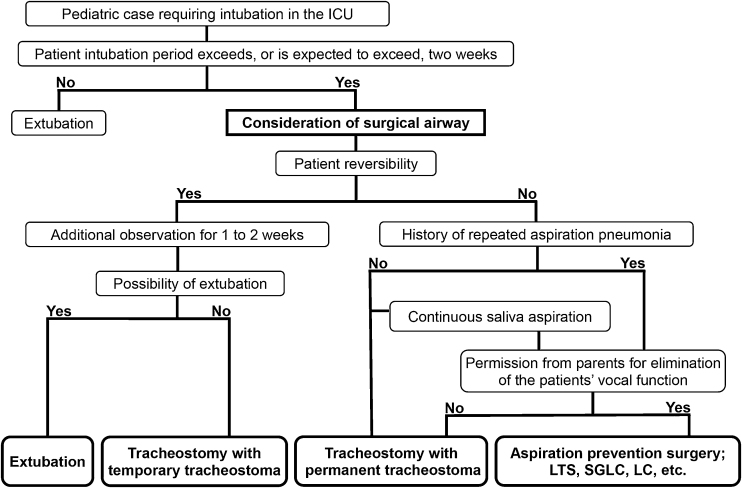
Flowchart for selecting a surgical airway. LTS, Laryngotracheal Separation; SGLC, Subglottic Laryngeal Closure; LC, Laryngeal Closure.

A flowchart for selecting an appropriate surgical airway for the long-term management of NIPP with intubation was created by a laryngologist and a pediatrician. Our flowchart includes the following main branching points; possibility of extubation, intubation period, reversibility related to prognosis, history of repeated aspiration pneumonia and/or continuous saliva aspiration, and permission from the parents for elimination of the patients’ vocal function (Fig. 1). The included surgeries were as follows: Tracheostomy with Temporary Tracheostoma (TwTT); Tracheostomy with Permanent Tracheostoma (TwPT), which includes sutures from the anterior wall of the trachea to the surrounding skin flaps^7^, ^8^; and APSs such as LTS.

#### Extubation

Referring to evidence-based guidelines for weaning and discontinuing ventilatory support,^9^ pediatricians decided the possibility of extubation. The decision was individually discussed. Extubation was considered if the following criteria were satisfied: evidence for some reversal of the underlying cause for respiratory failure; adequate oxygenation (eg, PaO_2_/FiO_2_ ratio > 150–200; requiring positive end-expiratory pressure [PEEP] ≤ 5–8 cm H_2_O; FiO_2_ ≤ 0.4–0.5); pH (eg, ≥7.25); hemodynamic stability, as defined by the absence of both myocardial ischemia and clinically significant hypotension (i.e., a condition requiring no vasopressor therapy or therapy with only low-dose vasopressors such as dopamine or dobutamine, <5 μg/kg/min); and the capability to initiate an inspiratory effort.

#### Intubation period

Meta-analysis reported that early tracheostomy (within 4–10 days) was associated with earlier weaning from mechanical ventilation, a shorter duration of sedation and reduced long-term mortality than late tracheostomy.^10^ However, early tracheostomy is particularly beneficial in selected adult patients, such as those with head or spinal cord injury or massive stroke.^11^, ^12^ In addition, early tracheostomy is not thought to be applicable to NIPP. The reversibility of pediatric patients is higher than that of adult patients, suggesting that more time to consider the indication of surgery is necessary. Another study of a nationwide survey reported that tracheostomies were performed during the first 14 days of mechanical ventilation in 409 (90%) of 455 ICUs.^13^ Referring to previous studies, we determined that the application of a surgical airway in a NIPP with intubation should be considered when the intubation period exceeds, or is expected to exceed, two weeks.

#### Reversibility

Reversibility related to prognosis was carefully determined after achieving consensus, following two or more discussions, during the intubation period by our multidisciplinary team, which included pediatric neurologists and intensivists. From the second discussion onward, each discussion was performed one week after the previous discussion. Reversibility was eventually determined when unanimous agreement was reached.

#### History of repeated aspiration pneumonia and/or continuous saliva aspiration

Aspiration has the potential to cause permanent damage to the developing lungs of infants and children. It is believed that children with dysphagia have a worse prognosis and are at a higher risk of repeated aspiration pneumonia, and aspiration in NIPP should be considered a signiﬁcant problem that affects prognosis.^6^ Therefore, we have included the following criteria in our flowchart; history of aspiration pneumonia and/or continuous saliva aspiration.

#### Permission from parents for elimination of the patients’ vocal function

Aspiration in NIPP is considered a signiﬁcant problem affecting mortality.^6^ Consequently, we included not only tracheostomy but also APS, as one of the ﬁrst-line treatments for airway management. However, vocal function is typically eliminated by a surgical procedure to prevent aspiration. Therefore, our team, consisting of a laryngologist, a pediatrician, and a nurse, obtain informed consent for the surgery from all parents whose children are candidates for APS with reference to publications in the American Academy of Pediatrics,^14^, ^15^ after conducting preoperative counseling and education.

### Study subjects

NIPP, defined as pediatric patients with disorders of the central nervous system resulting in motor impairment, cognitive impairment, and medical complexity, who required ventilator assistance, and who had been receiving prolonged intubation between 2017 and 2022 at Fukushima Medical University Hospital, were enrolled in this study.

A total of 21 subjects were included. The patient characteristics are described in Table 1. The selected surgeries were as follows: TwTT (n = 5); TwPT (n = 13); and APS (n = 3). All surgeries were performed safely without any perioperative complications such as intraoperative death or massive bleeding. Postoperative complications, such as accidental extubation or anastomotic leakage, were not observed, except in one case with tracheostomy stoma infection that required additional treatment. Following the procedure, 10 patients were able to return home and received home-based care such as mechanical ventilation. Four patients were transferred to a hospital that specializes in children with severe mental and physical disabilities. At the time of writing, three patients have been hospitalized for 97, 142, and 198 days after their respective procedures. Four patients could not be discharged, and died on Days 44, 113, 144 and 237 after their respective procedures because of exacerbations of their primary disease. Several months after initial TwPT, two patients underwent APS due to multiple aspiration pneumonia. Three of the five patients who underwent TwTT were successfully decannulated. At the time of writing, one of the remaining two patients was scheduled to undergo decannulation.

**Table 1:** Patient characteristics and selected surgeries.

Case	Age, yr	Sex	Height, cm	Weight, kg	Primary disease	Reason for intubation	Preoperative intubation period, days	Primary disease	Past history of AP or high risk of AP	Permission for elimination of VF	Selected surgery	Operation time, min	Bleeding, g	Patient outcome
1	2	F	84	13.5	Acute encephalopathy	Dysfunction of respiratory center	25	–	+	–	TwPT	44	0	Transferred to hospital
2	0	F	58.6	5.3	Severe perinatal asphyxia	Aspiration pneumonia	23	–	+	–	TwPT	55	0	Home-based care
3	2	F	73	9.5	Acute encephalopathy	Dysfunction of respiratory center	68	–	–	N/A	TwPT	53	0	Home-based care
4	2	M	78	9.2	Double outlet right ventricle	Congestive heart failure	358	+	–	N/A	TwTT	120	0	Home-based care
5	12	F	138.7	31	Aicardi syndrome	Aspiration pneumonia	19	–	+	–	TwPT	76	0	Transferred to hospital
6	16	M	118	16	Cerebral palsy	Status dystonicus	26	–	+	+	APS	194	10	Home-based care
7	0	M	68	7	Acute encephalopathy	Dysfunction of respiratory center	37	–	+	+	APS	214	0	Home-based care
8	0	F	57.5	3.6	Pfeiffer syndrome	Upper airway obstruction	28	–	+	–	TwPT	84	0	Died 237 days after the procedure
9	1	F	78	7.9	Hypoxic-ischemic encephalopathy	Dysfunction of respiratory center	8	–	+	+	APS	220	0	Home-based care
10	0	F	54.5	3.7	Trisomy 13	Respiratory failure	30	–	–	N/A	TwPT	75	0	Home-based care
11	6	F	110	15	Muscular dystrophy	Respiratory failure	25	+	–	N/A	TwTT	51	0	Home-based care
12	1	F	66	6.3	Severe perinatal asphyxia	Respiratory failure	138	–	–	N/A	TwPT	92	10	Died 144 days after the procedure
13	6	M	123	29	Acute encephalopathy	Respiratory failure	12	+	–	N/A	TwTT	66	0	Home-based care
14	1	M	72.5	7	Severe perinatal asphyxia	Dysfunction of respiratory center	71	–	–	N/A	TwPT	41	0	Transferred to hospital
15	2	F	85	12.1	Acute encephalopathy	Dysfunction of respiratory center	34	–	+	–	TwPT	54	0	Home-based care
16	0	F	58.5	4.9	Trisomy 13	Dysfunction of respiratory center	84	–	–	N/A	TwPT	83	0	Died 113 days after the procedure
17	3	M	91.6	13.9	Trisomy 18	Dysfunction of respiratory center	30	–	+	–	TwPT	92	5	Died 44 days after the procedure
18	0	M	70	7	Hypoxic-ischemic encephalopathy	Dysfunction of respiratory center	32	–	–	N/A	TwPT	80	0	Hospitalized for 198 days after the procedure
19	0	M	63.4	9.9	Infantile epilepsy	Dysfunction of respiratory center	69	+	–	N/A	TwTT	62	5	Transferred to hospital
20	1	F	75	8	Unilateral megalencephaly	Dysfunction of respiratory center	22	–	+	–	TwPT	72	0	Hospitalized for 142 days after the procedure
21	8	M	120	30	Acute encephalitis with refractory, repetitive partial seizures	Respiratory depression	40	+	–	N/A	TwTT	64	0	Hospitalized for 97 days after the procedure

AP, Aspiration Pneumonia; VF, Vocal Function; TwTT, Tracheostomy with Temporary Tracheostoma; TwPT, Tracheostomy with Permanent Tracheostoma; APS, Aspiration Prevention Surgery.

### Questionnaire surveys

First, to confirm the necessity of the flowchart, we conducted a survey of pediatricians regarding the selection of a surgical airway, which included interest and confidence in, as well as education regarding, surgical airway selection for prolonged-intubated children, and related comments, as shown in Table 2.

**Table 2:** Questionnaire survey for pediatricians.

1. Are you interested in how to select an appropriate surgical airway in pediatric intubated patients?
1. Yes
2. No

2. How confident are you in selecting an appropriate surgical airway in pediatric intubated patients?
1. Very confident
2. Confident
3. Unsure
4. Very unsure

3. Have you ever been educated or trained regarding appropriate selection of a surgical airway?
1. Yes
2. No

4. Have you ever engaged in the decision-making process regarding surgical airway selection?
1. Yes
2. No

5. Have you ever had to make a difficult decision regarding the appropriate selection of a surgical airway?
1. Yes
2. No

6. How satisfied were you with the decision-making process when using the flowchart?
1. Very satisfied
2. Satisfied
3. Dissatisfied
4. Very dissatisfied

7. How satisfied were you with the perioperative and postoperative course, including the selected surgical airway?
1. Very satisfied
2. Satisfied
3. Dissatisfied
4. Very dissatisfied

8. Please freely describe your perspectives regarding the selection of the appropriate surgical airway in pediatric intubated patients.

To evaluate our flowchart’s appropriateness, we also conducted questionnaire surveys of pediatricians and caregivers to investigate their satisfaction with the decision-making process using the flowchart, perioperative as well as postoperative course, and related comments, as shown in Table 2, Table 3. During the study period, all families of children were invited to participate, and decannulated patients were included. Referring studies in caregivers who care for pediatric tracheostomy patients,^16^, ^17^, ^18^ a questionnaire survey distributed to such caregivers was also conducted. Caregivers of children who remained hospitalized at the time of writing were excluded (n = 3). Four caregivers of children who could not be discharged and died in our hospital were also excluded. The parents of two patients declined to participate, leaving a study sample of 12. Ten primary caregivers completed the survey (the caregivers for Cases 2–7, 9–11, and 13). All 10 caregivers were female and were the mothers of the respective patients. The mean age was 40 years.

**Table 3:** Questionnaire survey for caregivers.

Did you feel distress/confusion during the decision-making process?
1. Yes
2. No
Did you feel that the burdens of the decision-making were reduced by the explanations from our team using the flowchart?
1. Yes
2. No
How satisfied were you with the decision-making process?
1. Very satisfied
2. Satisfied
3. Dissatisfied
4. Very dissatisfied
How satisfied were you with the perioperative and postoperative course, including the selected surgical airway?
1. Very satisfied
2. Satisfied
3. Dissatisfied
4. Very dissatisfied
Please freely describe your perspectives regarding the decision-making process.

## Results

### Survey of pediatricians regarding selection of a surgical airway

A survey was conducted on 14 pediatricians, eight males, with a mean age of 39 years. Seven pediatricians specialized in neonatology, three cardiology, two neurology, two in training. All pediatricians were interested in how to select the appropriate surgical airway for prolonged intubated children; however, nine of them did not have confidence to select an appropriate surgical airway, and seven had not been educated or trained regarding the appropriate selection of a surgical airway. Eleven pediatricians had experience selecting of surgical airways, nine of whom had had experiences in which they had to make a difficult decision. Free comments were as follows; “The risk of aspiration pneumonia is increased in seriously impaired pediatric patients when they grow older. Although the patients’ parents may have understood that surgery was preferable, they were significantly concerned about not being able to hear their children’s voice throughout the rest of their lives. Therefore, they find it difficult to make a decision”.

### Questionnaire survey for pediatricians and caregivers

#### Questionnaire survey for pediatricians

Fourteen pediatricians in charge of the patients who had undergone any of the procedures listed in our flowchart completed the questionnaire. The results of which showed that four pediatricians were very satisfied with the decision-making process, and ten were satisfied. Regarding the perioperative and postoperative courses that included the selected surgical airway, three pediatricians were very satisfied, and 11 were satisfied. None were dissatisfied or very dissatisfied with the process or course or care.

#### Questionnaire survey for care givers

The results of the questions regarding distress/confusion in the decision-making process showed that nine caregivers were distressed/confused. However, the burdens of seven caregivers were reduced after listening to the explanations provided by our team, who used the flowchart. The survey on satisfaction with the decision-making process showed that four caregivers were very satisfied, and six were satisfied. Regarding the perioperative and postoperative courses, including the selected surgical airway, five caregivers were very satisfied, and the remaining five were satisfied. No caregivers were dissatisfied or very dissatisfied with the decision-making process, postoperative course, or selected surgery.

### Case examples

Notable patient accounts decision-making process and postoperative course, showing each patient’s tracheostoma and the results of the questionnaire survey, are highlighted here for select patients.

#### TwTT case (Case 13)

**Figure 2:**
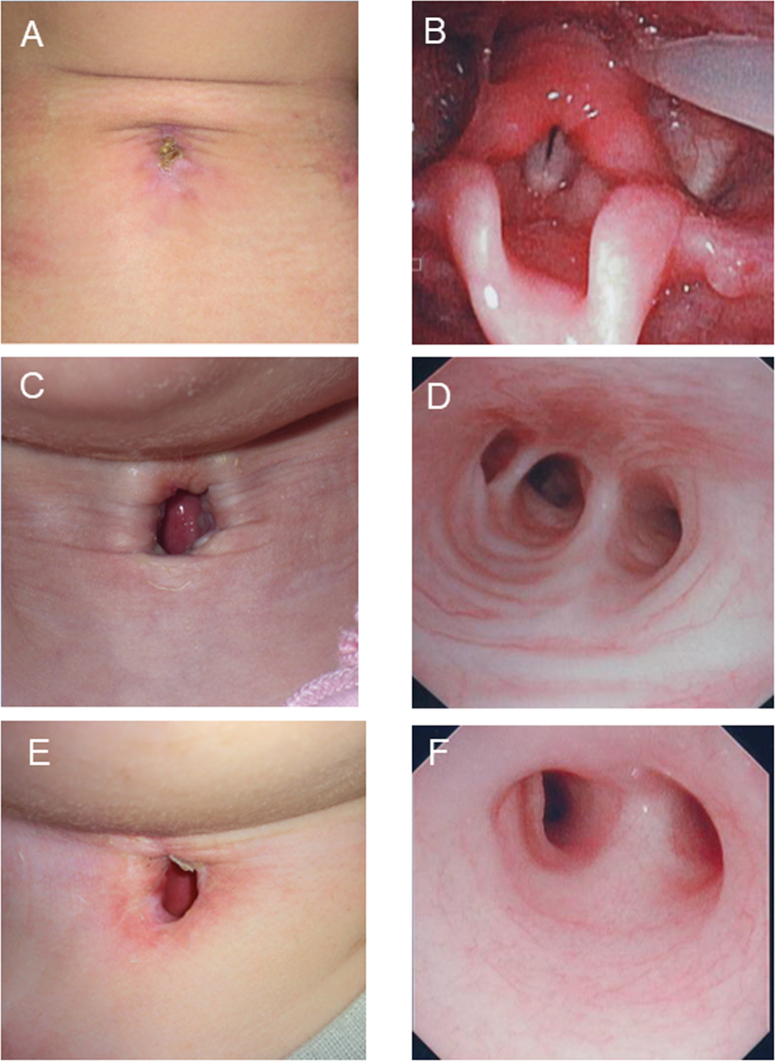
Findings of tracheostoma and trachea. (A) Successful decannulation was achieved 42 days after surgery in TwTT case. (B) Endoscopic findings showing no laryngeal stenosis in TwTT case. (C) Stable permanent tracheostoma without granulation tissue in TwPT case. (D) Endoscopic findings showing no tracheal stenosis in TwPT case. (E) Stable permanent tracheostoma without granulation tissue in APS case. (F) Endoscopic findings showing no tracheal stenosis in APS case.

The patient was a 6-year-old boy who was intubated for respiratory failure due to acute encephalopathy. He was diagnosed as having a reversible condition by his attending pediatricians; however, after 12 days the patient was still intubated, and it was predicted that intubation would continue beyond the 2-week point. Therefore, he underwent TwTT, 42 days after which he was successfully decannulated (Fig. 2A and B) and discharged from the hospital. The patient’s caregiver was distressed/confused during the decision-making process, but their decision-making burden was reduced after listening to explanations by our team, who used the flowchart, and they were very satisfied after the decision-making process was finished. The caregiver was also very satisfied with the perioperative and postoperative course.

#### TwPT case (Case 10)

The patient was a 5-month-old girl who was intubated for respiratory failure due to acute pneumonia. Her underlining conditions were trisomy 13 with an atrial septal defect, a ventricular septal defect, and patent ductus arteriosus. She was diagnosed as having an irreversible condition by her attending pediatricians. Her preoperative intubation period was 30 days. A history of repeated aspiration pneumonia was not confirmed. Therefore, she underwent TwPT. Ten months after the surgery, she was successfully discharged from the hospital and was able to receive home-based care with a stable permanent tracheostoma without tracheal stenosis (Fig. 2C and D). The patient’s caregiver was distressed/confused during the decision-making process, but their burden of making decision was reduced after listening to our explanations, and they were satisfied after the decision-making process was finished. The patient’s caregiver was very satisfied with the perioperative and postoperative course.

#### APS case (Case 9)

The patient was a 1-year-old girl who was intubated for dysfunction of the respiratory center due to hypoxic-ischemic encephalopathy. She was diagnosed as having an irreversible condition by her attending pediatricians. The preoperative intubation period was 8 days; however, this period was expected to be more than two weeks. A history of repeated aspiration pneumonia was confirmed. Informed permission for the elimination of vocal function was obtained from her parents. Therefore, she underwent APS. One month after the surgery, she was successfully transferred to a local hospital and was finally able to receive home-based care with a stable permanent tracheostoma without tracheal stenosis (Fig. 2E and F). The patient’s caregiver was distressed/confused during the decision-making process, but their burden of making decision was reduced after listening to our explanations and they were satisfied after the decision-making process was finished. The caregiver was also satisfied with the perioperative and postoperative course.

## Discussion

Inappropriate airway management in ventilated intubated children, including incorrect timings and methods, can lead to worsening of respiratory condition, and may cause laryngeal and/or tracheal stenosis, as well as an increase in mortality. In the current study, all pediatricians were highly motivated to learn how to select the appropriate surgical airway for prolonged intubated children; however, many were not confident when it came to appropriate surgical airway selection. Studies have reported the indications of tracheostomy for pediatric^3^, ^19^, ^20^ and adult patients^4^, ^10^; however, there has been no report to date for appropriate surgical airway selection in NIPP with intubation with the use of a flowchart. Our flowchart was originally designed for a pediatrician who first has to propose a surgical airway to the parents of NIPP with intubation. Without defined criteria, the surgery may be selected subjectively by the pediatricians and/or caregivers, using their own ethics and experience. We believe that our flowchart will be of significant help. By referring to the current study, not only pediatricians and parents who care for the patients but also pediatric otolaryngologists who perform the surgery, may have their burdens eased and confusion regarding the decision-making process reduced.

We selectively performed TwTT or TwPT because the optimal timing of decannulation varies among patients, depending on indications and pathological conditions. The decannulation rate in patients with neurologic disorders has been demonstrated to be significantly lower than that in patients with trauma.^3^ In addition, care of a permanent tracheostoma is easier than that of a temporary tracheostoma.^7^, ^8^ In the current study, three of the five patients who underwent TwTT were successfully decannulated, one patient was scheduled to undergo decannulation, and all patients who underwent TwPT could not be decannulated, which may indicate the appropriateness of our flowchart.

A single surgical procedure is ideal, especially in an irreversible severely impaired pediatric case; thus, we included APS in our flowchart. A variety of surgical approaches for aspiration, such as LTS, laryngeal closure and total laryngectomy, have been reported. Among these surgeries, LTS is a theoretically reversible procedure, and is preferred by many physicians for use in children.^6^, ^21^, ^22^, ^23^ However, few successful reversals have been reported.^24^ In the current study, therefore, APS including LTS was treated as a procedure that eliminates the vocal function of NIPP. Two patients (Cases 2 and 20), who had undergone TwPT several months before, underwent APS. However, for these patients, in whom APS was considered, for which the flowchart was used, informed permission regarding elimination of vocal function was not obtained from their parents. As anticipated, these patients experienced multiple aspiration pneumonia after TwPT. In terms of accurate prediction of prognosis, the above-mentioned cases might prove the appropriateness of inclusion of APS in the flowchart. Although both patients’ parents were satisfied with the decision-making process and the present circumstances, further discussion is required regarding more appropriate criteria.

There are several limitations to the current study. First, we here demonstrated the satisfaction of caregivers; however, questionnaire surveys were not obtained from all caregivers. This is because we excluded caregivers of children who in our hospital. We assumed it would be difficult for these caregivers to express negative opinions without emotion, and our focus was the caregivers’ feelings regarding the postoperative course after leaving the hospital. Though our case series demonstrated an importance of the perspectives of caregivers, small numbers of questionnaire respondents might have affected our results. Studies of a similar design but with larger study populations would solve this limitation. Second, we did not perform long-term follow-up on the satisfaction of the caregivers or the pediatricians. Since the purposes of the creation of our flowchart were to reduce the burden on pediatricians and caregivers, as well as increase their satisfaction with the decision-making process, long-term surveys of their satisfaction might provide us with additional meaningful information.

## Conclusion

The present study demonstrated the applicability of a flowchart for selecting an appropriate surgical airway in NIPP with prolonged intubation. Using our flowchart, pediatricians and caregivers are likely to be able to select an appropriate surgical airway, leading to increased satisfaction with the decision-making process and postoperative course.

## Funding

The authors have no funding, financial relationships, or conflicts of interest to disclose.

## Conflicts of interest

The authors declare no conflicts of interest.
